# The Concentration of Vanadium in Pathologically Altered Human Kidneys

**DOI:** 10.1007/s12011-017-0986-2

**Published:** 2017-03-09

**Authors:** Aleksandra Wilk, Barbara Wiszniewska, Dagmara Szypulska-Koziarska, Paulina Kaczmarek, Maciej Romanowski, Jacek Różański, Marcin Słojewski, Kazimierz Ciechanowski, Małgorzata Marchelek-Myśliwiec, Elżbieta Kalisińska

**Affiliations:** 1 Department of Histology and Embryology, Pomeranian Medical University, Al. Powstańców Wielkopolskich 72, 70-111 Szczecin, Poland; 2 Department of General Surgery and Transplantation, Pomeranian Medical University, Al. Powstańców Wielkopolskich 72, 70-111 Szczecin, Poland; 3 Department of Nephrology, Transplantology and Internal Medicine, Pomeranian Medical University, Al. Powstańców Wielkopolskich 72, 70-111 Szczecin, Poland; 4 Department of Urology and Urological Oncology, Pomeranian Medical University, Al. Powstańców Wielkopolskich 72, 70-111 Szczecin, Poland; 5 Department of Biology and Medical Parasitology, Pomeranian Medical University, Al. Powstańców Wielkopolskich 72, 70-111 Szczecin, Poland

**Keywords:** Vanadium, Kidney, Kidney cortex, Kidney graft, Kidney medulla, Renal cell carcinoma

## Abstract

Vanadium has a unique and beneficial effect on both humans and animal organisms; however, excessive amount of the above-mentioned metal can cause many alterations in tissues and organs, including the kidneys. The aim of the study was to determine the concentration of vanadium (V) in the kidneys removed from patients due to lesions of various etiologies, including the rejection of the transplanted kidneys. Additionally, we determined the influence of selected biological and environmental factors on the V concentration. The study material consisted of the kidneys with tumor lesions (*n* = 27) and extracted kidney grafts (*n* = 10) obtained from patients from the north-western Poland. The V concentrations were assessed by atomic absorption spectrophotometry emission in inductively coupled argon plasma and expressed in concentrations in dry weight (dw). Statistically significant differences were observed for V concentrations in the renal medulla between the kidneys with tumors and renal grafts, where the lowest concentration of V was observed. The kidneys in more advanced stages of the tumor (T3 + T4) contained more vanadium than the kidneys of T1 + T2 stages and medians were 2.07 and 1.51, respectively. We also compared the V concentration in the kidneys between the renal grafts (K2) and the kidneys with tumor (K1) in two stages of advancement: T1 with T2 (K1_1 + 2_) and T3 with T4 (K1_3 + 4_). Statistically significant differences were noted between the renal medullae of the above-mentioned groups of kidneys.

According to the previous studies on the concentrations of other heavy metals, renal grafts accumulate less vanadium than cancerous kidneys, what can be associated with the immunosuppressive drugs taken by patients after the transplantation.

## Introduction

Vanadium, a transition metal having a unique and beneficial effect on both humans and animals, finds its primary uses in the metallurgy and in aerospace, as well as in chemical, glass, and photographic industries [1]. So far, studies have shown that vanadium is an essential trace element, of which only about 10 μg is required per day. Vanadium in the diet is found in unrefined oils, soy bean oil, olive oil, peanut oil, cottonseed oil, black pepper, mushrooms, parsley, spinach, and shellfish [2]. Its deficiencies can lead to many different pathological changes, inter alia growth limitations, pathologies of teeth and bone calcification, and of the reproductive system [3]. However, according to the International Agency for Research on Cancer (IARC), vanadium pentoxide is thought to be carcinogenic in humans and has been already classified as a group 2B carcinogen (indicating limited evidence of carcinogenicity in humans and less than sufficient evidence of carcinogenicity in experimental animals).

Furthermore, excessive amount of vanadium can cause many alterations in tissues and organs, including the kidneys which are particularly sensitive to this metal. The mechanism of its toxicity has not been fully understood yet. Nevertheless, it is believed that vanadium induces oxidative stress that results in, among other problems, lipid peroxidation, DNA degeneration, and denaturation of proteins, ultimately leading to the disintegration of the cell membranes [4]. The harmful effects of vanadium depend on the way of administration and the chemical form: its toxicity increases with valence, and the pentavalent forms (e.g., V_2_O_5_) seem to be the most harmful [1, 5, 6].

The kidneys are critical to the action of vanadium and studies on its nephrotoxic activities have been carried out for years, mainly on animal tissues. It is known that the exposition to too high concentration of the above-mentioned metal negatively affects various structures of the kidneys, including glomerulonephritis [7]. Furthermore, intraperitoneal administration of vanadium to the animals provoked acute glomerulonephritis with partial tubular and glomerular necrosis associated with acute renal failure. Vanadium also induces hypertension, which indeed is connected with renal dysfunction. In addition, this metal disrupts the biochemical and electrolyte balance [8].

Renal cell carcinoma (RCC) accounts for 90–95% of all kidney malignancies [9], and it mostly consider the renal cortex and infrequently in the medulla [10–12]. Over the last few decades, RCC has accounted for about 2% of all malignant tumors in humans [13]. The frequency of detection and the probability of occurrence of renal cell carcinoma are greater in highly industrialized countries than those in developing countries. In the USA, death rates from RCC have increased during the last 30 years by 35% in men and by 20% in women [14]. This may be related both to the adverse impact of environmental pollutants, including heavy metals, as well as visual examination and highly developed diagnostic.

Many research centers conduct studies on the concentrations of heavy metals and micro and macroelements in the kidneys, focusing on tumor lesions. The main aim of the above-mentioned studies was to determine the effects of selected metals on the process of carcinogenesis and changes, in the proportions and relationships between nephrotoxic and essential elements present in kidneys. It is believed that the kidneys of patients with cancer acculumate less Cd and more Pb [15–17]. Accordingly, to the vanadium, its concentration in pathologically altered kidneys versus healthy kidneys is controversial. However, little is known about the concentrations of the above-mentioned element in the human kidneys, especially separately in the renal cortex and renal medulla.

The aim of current study was to determine the concentrations of vanadium in the kidneys removed due to renal cancer and from patients with early rejection of kidney grafts. In addition, the study evaluated the effect of selected biological and environmental factors on the concentrations of this toxic metal.

## Materials and Methods

The research was carried out between 2009 and 2011. Material consisted of kidneys from patients hospitalized at the Department of Urology and General Surgery and Transplantation of the Independent Public Clinical Hospital No. 2 at the Pomeranian Medical University in Szczecin, north-western Poland. The examined organs came from women and men ranged 21 to 70 and 28 to 76 years, respectively. The patients were divided into two groups according to age: <50 years old and ≥50 years old. Additionally, they were divided into two groups according to place of residence: cities with population over and below 100,000. All patients came from north-western Poland.

The 37 samples of kidneys were derived from patients following nephrectomy due to the presence of tumors (*n* = 27) and from kidney grafts from patients following transplantation due to kidney failure (*n* = 10). The study was approved by the Bioethics Committee of the Pomeranian Medical University (Resolution No. KB-0080/61/09).

The kidneys were separated into the cortex and medulla. The samples were dried to constant weight at 105 °C for the determination of vanadium. Dried samples were ground in an agate mortar. The samples were wet mineralized by wet digestion using a Velp Scientifica mineralizer (Usmate Velate, Italy) in a mixture of concentrated HNO_3_ and HClO_4_ (Suprapur Merck®) [18].

The V determinations were performed using inductively coupled plasma atomic emission spectrophotometry (ICP AES), on a Perkin-Elmer Optima 2000 DV. The correctness of the analysis was controlled by determination of the analyzed elements in reference material of a known concentration. The average percentage of water content in either part of the kidney was similar at about 80%. The concentrations of the vanadium were expressed as milligrams per kilograms dry weight (dw).

Statistical analysis used Stat Soft Statistica 9.0 software and Microsoft Excel 2007. Arithmetic mean (*X*), standard deviation of *X* (SD), and median (*M*) were established for the concentrations of the analyzed element. To evaluate the compliance of the results with the expected normal distribution, Kolmogorov-Smirnov (K–S) tests with Lilliefors correction were used (*p* < 0.05). In addition, mean concentrations in the corresponding parts of the kidney were compared between those of the different patient groups. As the data distribution was not consistent with the expected normal distribution, Kruskal-Wallis (K–W) tests and Mann-Whitney *U* tests (M–W *U*; *p* < 0.05) were used.

## Results

The reliability of the analytical procedure was controlled by the determination of vanadium in reference material with known concentrations: DOLT 4 Dogfish liver (National Research Council Canada). The application of the method to the quantification of vanadium in the reference material gave 104% recovery.

The K–S test, with and without the Lilliefors correction, revealed no characteristics of normal distribution. The mean concentrations of metal in the samples were therefore compared using a nonparametric M–W *U* test.

No statistically significant differences in the concentration of vanadium between the cortical and medullar region of any of the examined kidneys were observed and the concentrations of V were 1.91 and 1.97 mg/kg dw, respectively (Table 1). Additionally, no significant differences were seen when comparing the concentration of vanadium across sex or age groups (less than 50) (A1) and more than 50 years old (A2) (Table 1). No significant differences were noted between place of residence (taking into account cities with population over and below 100,000) and V concentration (Table 2).

**Table 1:** The concentration of vanadium (in mg/kg dw) in the renal cortex and renal medulla

Examined parameter	Cortex	Medulla
	All patients	
*N*	37	37
*X* ± SD	1.91 ± 2.98	1.97 ± 1.96
Median	0.84	1.34
Min–max	0.24–15.28	0.23–9.04
	Females	
*N*	17	17
*X* ± SD	1.39 ± 1.47	2.17 ± 2.51
Median	0.63	1.55
Min–max	0.23–5.64	0.23–9.04
	Males	
*N*	20	20
*X* ± SD	2.35 ± 3.81	1.80 ± 1.39
Median	0.99	1.22
Min–max	0.29–15.28	0.32–5.27
	A1	
*N*	9	9
*X* ± SD	0.92 ± 0.85	1.11 ± 0.86
Median	0.59	0.83
Min–max	0.29–2.73	0.31–2.91
	A2	
*N*	28	28
*X* ± SD	2.23 ± 3.34	2.25 ± 2.14
Median	0.99	1.44
Min–max	0.23–15.28	0.23–9.04

*N* number of patients, *X* arithmetical mean, *SD* standard deviation, *A1* patients <50 years of age, *A2* patients >50 years

**Table 2:** Comparison of vanadium concentration (in mg/kg dw) in the renal cortex and medulla between residential groups

Examined parameter	B1		B2	
	Cortex	Medulla	Cortex	Medulla
*N*	19	26	11	11
*X* ± SD	2.26 ± 3.49	1.92 ± 2.01	1.3 ± 1.61	1.88 ± 2.14
Median	1.04	1.44	0.6	1.11
Min–max	0.23–15.28	0.32–9.04	0.23–5.64	0.24–7.24

*N* number of patients, *X* arithmetical mean, *SD* standard deviation, *B1* residents of cities with population over 100,000, *B2* residents of cities below 100,000

In order to determine whether there were differences in the values ​​of V concentration between the kidneys with tumors (K1) and grafts (K2), separately for the cortex and the medulla of the kidney, the M–W *U* test was used. The median V concentrations in the renal medulla of tumor kidneys and renal grafts were 1.57 and 0.41 mg/kg dw, respectively, and the difference was statistically significant (*p* = 0.0005). Comparing K1 and K2, the smallest V concentration was found in the cortex of the renal grafts at 0.36 mg/kg dw (Table 3).

**Table 3:** The concentration of vanadium (in mg/kg dw) in the cortex and the medulla of the cancer kidney (K1) and renal graft (K2)

Examined parameter	Cortex		Medulla	
	K1	K2	K1	K2
*N*	27	10	11	10
*X* ± SD	2.41 ± 3.35	0.55 ± 0.5	2.44 ± 2.06	0.69 ± 0.79
Median	1.1	0.36	1.57	0.41
Min–max	0.24–15.29	0.23–1.92	0.24–9.04	0.29–2.91

*N* number of patients, *X* arithmetical mean, *SD* standard deviation, *M–W U* Mann-Whitney *U* test, *p* level of significance, *NS* not significant

Additionally, the V concentration in the kidneys was compared between the renal grafts (K2) and the kidneys with tumor (K1) in two stages of advancement: T1 with T2 (K1_1 + 2_) and T3 with T4 (K1_3 + 4_). Statistically significant differences were noted between the renal medullae of above-mentioned groups of kidneys (K2 versus K1_1 + 2_), 0.41 versus 1.51 mgV/kg dw and (K2 versus K1_3 + 4_), 0.41 versus 2.07 mgV/kg dw, *p* < 0.05. Comparing the two groups of kidneys with tumor (K1_1 + 2_ versus K1_3 + 4_), the median V concentration was 27% higher in the kidneys with more advanced stages of cancer compared to that of the kidneys with less tumor stages, but differences were not statistically significant.

## Discussion

Although studies have been conducted on vanadium for many years, little data is still available. Most of nephrotoxicological research is based on other heavy metals, such as cadmium, mercury, and lead [19–24]. What is more, the articles in the literature mostly refer to animal studies. The available human studies generally concern the entire kidney or renal cortex and do not specifically take the medullary region into consideration. It is believed that the cortical region of the kidney is more prone to accumulation of certain elements. It is due to the localization of the renal corpuscles which are responsible for the blood filtration and the production of the primary urine.

In the present study, larger vanadium concentrations were found within the medulla (1.34 mg/kg) than those in the cortex (0.84 mg/kg), for all the examined kidneys; however, the difference was not statistically significant. In the study conducted by Wilk et al. [19], the lead concentration was also significantly higher in the medullary region than that in the cortex ~0.3 and ~0.5 mg/kg, respectively. It seems that distribution of the aforementioned metals is related with affinity of these metals to specific structures of the kidney, including the Henle’s loop [19]. There is thus a need to conduct additional nephrotoxicological researches on heavy metal concentrations, considering the cortical and medullary regions separately.

Nephrotoxicological studies also concern the influence of biological factors on the distribution of heavy metals in the kidney. The age of subjects is a significant biological parameter in analyzing the toxic metals concentrations in the kidneys. In the current study, no correlation between the sex, age, and concentration of vanadium in all examined kidneys was confirmed, whereas other authors reported changes in trace element concentrations depending on age [20, 25].

Apart from biological factors, the concentration of heavy metals in the kidneys depends on the condition of the organ, including the type and stage of disease [15, 17, 19]. It is believed that kidneys with neoplastic changes accumulate less Cd but more Pb [15, 17, 25]. Accordingly, to the vanadium, its concentration in pathologically altered kidneys versus that of healthy kidneys is controversial. Studies conducted by Kwiatek et al. [16] have shown that the concentrations of all of trace elements, including vanadium, were lower in cancerous kidneys (3 mg/kg) than those in the control group (9.8 mg/kg). Whereas, Dobrowolski et al. [15] showed that vanadium concentration of the cortices of healthy kidneys was lower than that in cancerous kidneys; 0.5 and 0.9 mg/kg, respectively (Table 4). The present study’s values for the concentration of vanadium in the cortex (1.1 mg/kg) of the kidneys with tumors correspond with the value achieved by Dobrowolski et al. [15]. This is probably due to the progressive degradation of the organ, since all the experimental kidneys were pathologically altered. However, it should be mentioned that the concentration of heavy metals, including vanadium, may be influenced by environmental and biological factors. It is worth noting that Kwiatek et al. [16] did not take into account the division of the kidney into the cortex and the medulla, which may thus explain the different results in the various studies. It is thus of special importance to separately examine the cortical and medullary regions of the kidney.

**Table 4:** The concentration of vanadium (in mg/kg dw) in human kidneys in other studies

Part of the kidney	Type of the kidney	V concentration	Author
C + M (*N* = 15)	healthy renal tissue	9.8	Kwiatek et al. [16]
C + M (*N* = 34)	kidney with tumor	3.0	Kwiatek et al. [16]
C (*N* = 15)	healthy renal tissue	0.5	Dobrowolski et al. [15]
C (*N* = 36)	kidney with tumor	0.9	Dobrowolski et al. [15]
C (*N* = 27)	kidney with tumor	0.84	Present study
C (*N* = 10)	renal graft	0.36	Present study
M (*N* = 11)	kidney with tumor	0.34	Present study
M (*N* = 10)	renal graft	0.41	Present study

*C* cortex, *M* medulla

Due to the intake of immunosuppressive drugs of recipients, grafts are gradually degradated, which finally leads to organ rejection. The current study concerns the vanadium concentration in rejected kidneys. No significant differences in vanadium levels were found between the cortices of the grafts (0.36 mg/kg) and the cortices of cancerous kidneys (1.1 mg/kg). However, significant differences existed in the medullae (*p* = 0.005). The vanadium concentration in the medullae of the grafts and cancerous kidneys was 0.41 and 1.57 mg/kg, respectively. Similar results were shown in the experiment performed by Wilk et al. [19], where the concentrations of cadmium, lead, and mercury were lower in renal grafts than those in the cancerous kidneys. This is probably due to having fewer properly functioning nephrons in the transplanted organs, since the nephrons are damaged during transplantation. Another possible explanation for lower accumulation of heavy metal in the grafts is perhaps the use of immunosuppressive medicines by renal transplant patients. It may be that immunosuppressive drugs decrease the concentration of heavy metals in some organs, including the kidney. Due to the highly statistically significant difference between the studied groups, the rejected kidneys were compared with the cancerous kidneys in different stages of advancement. These differences were also significant.

The following studies have shown how extremely important is to consider the content of heavy metals in the cortex and the medulla of the kidney separately. The results concerning the current study are statistically significant only within the renal medulla, where the correlation between the concentration of vanadium and the stage of cancer and between renal grafts and cancerous kidneys was noted. The data also represent an important reference point for future research on the concentration of heavy metals in organs.

In summary, vanadium in rejected and cancerous kidneys tends to accumulate in higher amount in the renal medulla than in cortex (1.34 and 0.84 mg/kg dw, respectively), what can be explained by individual affinity of vanadium to the structures of the kidney, including the Henle’s loop that are only within the renal medulla and/or its specific transport proteins. Renal grafts accumulate less vanadium than cancerous kidneys (the renal cortex: 0.36 versus 1.1 mg/kg dw; the renal medulla: 0.41 versus 1.57 mg/kg dw) independently on the stage of advancement, what could have been caused by immunosuppressors taken by the graft recipients.
